# Influence of urban river restoration on nitrogen dynamics at the sediment-water interface

**DOI:** 10.1371/journal.pone.0212690

**Published:** 2019-03-13

**Authors:** Anna M. Lavelle, Nic R. Bury, Francis T. O’Shea, Michael A. Chadwick

**Affiliations:** 1 Department of Geography, King’s College London, London, United Kingdom; 2 School of Science, Technology and Engineering, University of Suffolk, Ipswich, United Kingdom; 3 Suffolk Sustainability Institute, University of Suffolk, Ipswich, United Kingdom; Universitat de Barcelona, SPAIN

## Abstract

River restoration projects focused on altering flow regimes through use of in-channel structures can facilitate ecosystem services, such as promoting nitrogen (N) storage to reduce eutrophication. In this study we use small flux chambers to examine ammonium (NH_4_^+^) and nitrate (NO_3_^-^) cycling across the sediment-water interface. Paired restored and unrestored study sites in 5 urban tributaries of the River Thames in Greater London were used to examine N dynamics following physical disturbances (0–3 min exposures) and subsequent biogeochemical activity (3–10 min exposures). Average ambient NH_4_^+^ concentrations were significantly different amongst all sites and ranged from 28.0 to 731.7 μg L^-1^, with the highest concentrations measured at restored sites. Average NO_3_^-^ concentrations ranged from 9.6 to 26.4 mg L^-1^, but did not significantly differ between restored and unrestored sites. Average NH_4_^+^ fluxes at restored sites ranged from -8.9 to 5.0 μg N m^-2^ sec^-1^, however restoration did not significantly influence NH_4_^+^ uptake or regeneration (i.e., a measure of release to surface water) between 0–3 minutes and 3–10 minutes. Further, average NO_3_^-^ fluxes amongst sites responded significantly between 0–3 minutes ranging from -33.6 to 97.7 μg N m^-2^ sec^-1^. Neither NH_4_^+^ nor NO_3_^-^ fluxes correlated to sediment chlorophyll-*a*, total organic matter, or grain size. We attributed variations in overall N fluxes to N-specific sediment storage capacity, biogeochemical transformations, potential legacy effects associated with urban pollution, and variations in river-specific restoration actions.

## Introduction

The “urban stream syndrome” provides a framework for evaluating changes associated with urbanization [1–5], including physical habitat modifications, hydrological alterations, and elevated nutrient loads occurring in catchments across the globe [6,7]. In urban environments, impervious surface cover and channel impoundments can off-set hydrologic connectivity between the stream channel, hyporheic, and riparian zones, resulting in complex sediment-supply dynamics [8,9]. In addition, altered flow regimes can modify ecological function [6], including nitrogen cycling [10–12] which can be compounded by elevated nutrient loads from gutters and storm drains [2,13,14]. ‘Urban karsts’, encompassing a complex, predominantly hidden, network of buried headwaters streams, sewers, and potable water pipes can further modify hydrological processes, reducing water infiltration and inhibiting nutrient storage capacity [12,15]. Together these factors can play a major role in influencing nitrogen dynamics in urbanised river ecosystems.

The presence of nitrogen in urban rivers is a major management issue due to high inputs from runoff and groundwater contamination [16]. Recent studies have estimated that anthropogenic N from grey water footprints can contribute up to 32.6 million tonnes per year to freshwater systems [17], resulting in widespread problems with eutrophication and hypoxia [18] In addition, urban watersheds receive N inputs from indirect sources, such as atmospheric deposition, diffuse land-based practices (e.g., fertilizers), unregulated discharges, leaky septic pipes, and misconnections [12,19,20]. In the Thames catchment, NO_3_^-^ concentrations have been reported in ranges between ~5 to ~35 mg L^-1^ [21,22], whilst NH_4_^+^ has been noted between ~100 to ~700 μg L^-1^ [23,24]. These concentrations from highly urban environments differ significantly from lower N concentrations observed in more rural UK rivers (<100 μg L^-1^) [23].

Sediment nitrogen dynamics (i.e., uptake, net movement into sediments, and regeneration, net movement into the water column) via physical and biogeochemical processes are influenced by a wide range of factors, including river discharge, sediment type, water quality, and stream metabolism [25–29]. NO_3_^-^ in particular is highly abundant in urban rivers and subject to assimilation, storage and denitrification via algae, aquatic plants, and microbes [16,30]. N is also known to control and limit Chl-*a* concentrations in urban systems [21], whilst also being influenced by sediment type and quality and quantity of organic matter [20]. The physical and biogeochemical processes that influence such N dynamics are predominantly focused at the sediment-water interface, and more investigations of these ecosystems functions are needed in urban streams and rivers [16].

To date, a handful of studies have examined the implications of river restoration on N processing [30–32]. Most restoration practises have focused on improving hydromorphology rather than modifying biogeochemical processes [32]. However, recent approaches have considered how habitat engineering focused on geomorphic stabilization, hydrologic connectivity, and flow manipulations (e.g. creating debris dams, backwaters, and eddies) can influence N dynamics (including nitrification and ammonification) via uptake and regeneration [19,33,34]. Additionally, modifying flow regime can encourage sediment organic matter retention and hyporheic anoxia due to increased heterotrophic respiration and prolonged contact time with denitrifying bacteria [19,35,36]. Further links have also been made between restoration activity, uptake lengths [37], and increased N ion retention capacities, which can result in nutrient reductions further downstream [16,38]. Due to the need for greater understanding of N biogeochemical processes following river restoration, the aim of this study was to determine how restoration of urban streams influences patch-scale N dynamics at the sediment-water interface. We hypothesized that urban river restoration should affect N uptake across the sediment-water interface. This was achieved through the use of a sediment-water interface assay to quantify NH_4_^+^and NO_3_ fluxes, defined as either uptake from the water column into the sediment or regeneration from the sediment into the water column, in restored and unrestored sites of tributaries of the River Thames, Greater London, UK.

## Material and methods

### Study area

**Fig 1:**
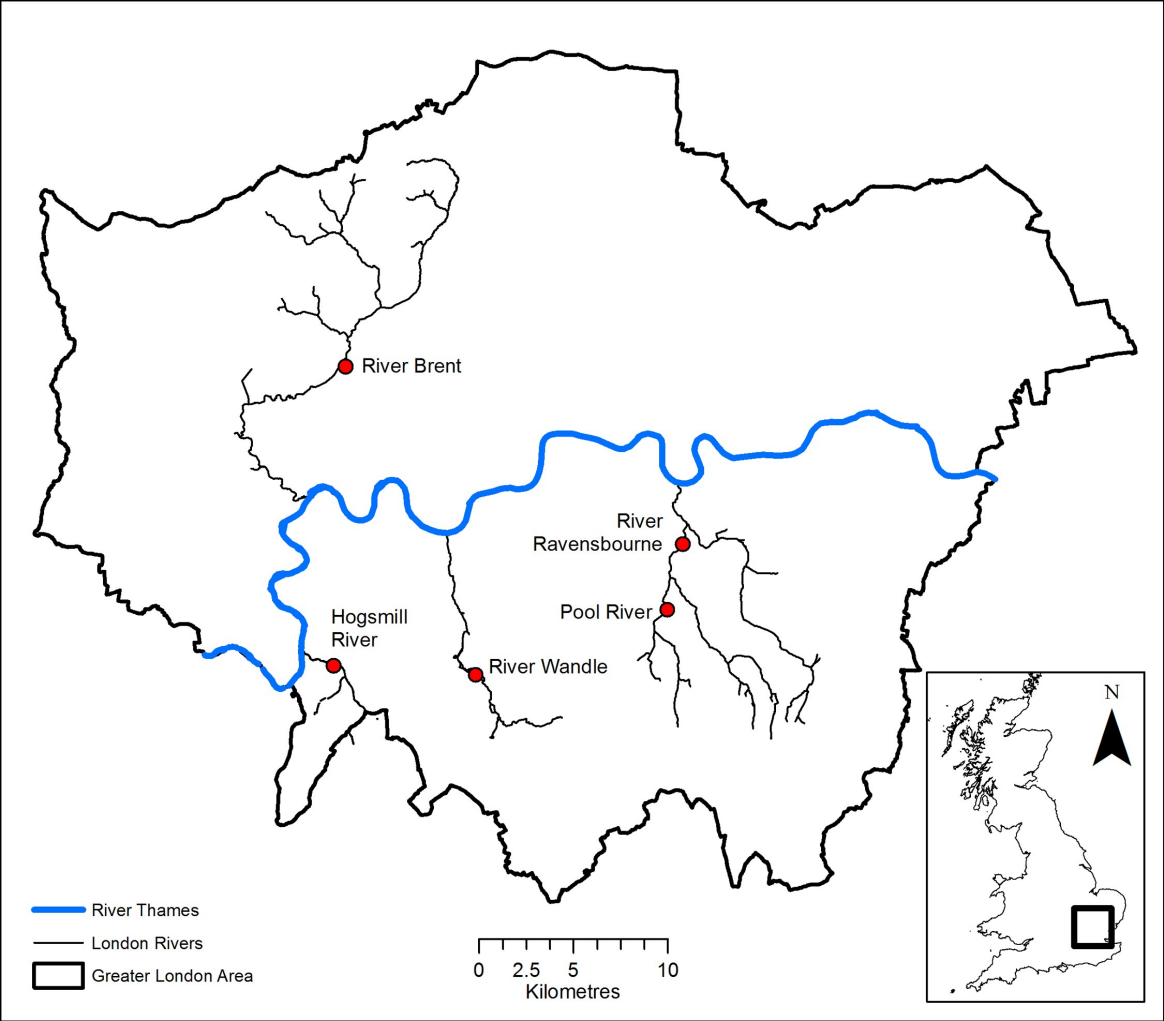
Study sites situated within Greater London, UK. Dots highlight the locations of each of the five study rivers, Ravensbourne, Pool, Wandle, Hogsmill and Brent.

Five paired restored and unrestored sites from urban tributaries of the River Thames in Greater London were selected from the River Restoration Centre database (Fig 1 and Table 1). These sites, used in previous research [39], comprised 25 meter long reaches which varied in terms of urban cover, land use and restoration approaches, [39]. On the river Brent and Wandle the restored reach was downstream, whereas on the Pool, Ravensbourne, and Hogsmill the restored reach was upstream. In all the study rivers, the reaches examined were approximately 50 – 250m apart. Hydrogeomorphological features were characterized by low gradient and shallow beds (<0.5 m), non-turbulent flows and underlying geology dominated by chalk and/or sandstone. Land use was predominantly urban, owing to high density housing within each catchment boundary. Historic channel straightening, culverting, and industrial activities (i.e., mills) had previously led to concerns over flooding, contamination, and functional connectivity across these river networks [39,40].

**Table 1:** Characteristics of restoration among the study rivers, including total river length (km), % urban (total urban land cover for the study river catchment), restoration project with completion year of the project in parenthesis. Data for this table are from Smith and Chadwick [39].

River	Site	River Length	% Urban	Urban pressures	Restoration
Ravensbourne (2008)	Ladywell Fields	18	51	Channelization & culverting	Re-meandering through parks
Pool (2012)	Bell Green	5	57	culverting, vegetation & fish loss	berms & redirecting flows
Wandle (2015)	Carshalton	14	47	Impoundment, weirs, low flow & oxygen levels	Lowering of weir & shortening fish passages
Hogsmill (2014)	Green Lane	10	39	Fish pass obstructions, weirs & sewage	Weir removals, creation of pools & riffles, channel narrowing
Brent (2003)	Tokyngton Park	29	69	Impoundments & habitat degradation	Recycling of concrete, re-meandering & creation of backwaters

Restoration efforts within the study rivers (Ravensbourne, Pool, Wandle, Hogsmill, and Brent) have primarily focused on restoring heterogeneous flows, hydrological connectivity, and habitat biodiversity (Table 1). Additional re-meandering structures have been engineered at the Pool to mitigate against the effects of historic gas work contamination [39]. The Wandle is of particular note, where the implementation of inadequate fish passages and barriers have impeded longitudinal connectivity [41]. Combined with storm water inputs from sewage works, this has triggered sediment deposition, nutrient loading, and oxygen depletion [41]. In response, restoration efforts have been made to counteract problems associated with weirs and concrete beds by re-naturalizing flows. At the Brent, flood and pollution preventative approaches have been taken to deploy willow poles and re-cycle ground concrete to generate riffle pools and encourage habitat stabilization. The creation of backwaters has led to the succession of new habitats, acting as a buffer zone during pollution and flood events [42].

### NH_4_^+^ and NO_3_^-^ flux assays

**Fig 2:**
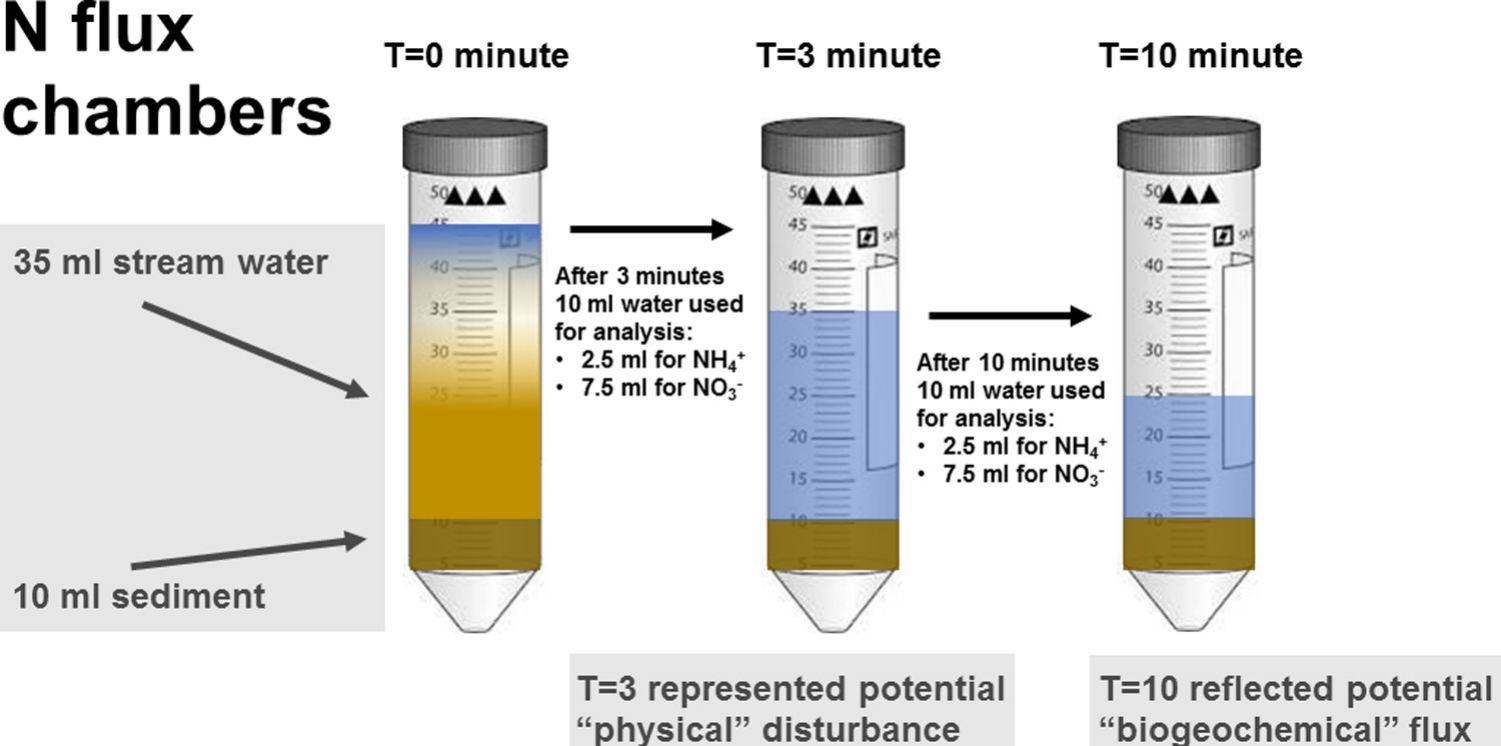
Experimental flux chambers: 10 mL sediment from the benthic zone were randomly collected, transferred into separate 50 mL falcon tubes and mixed with 35 mL stream water. For N samples, 10 mL water (2.5 mL for NH_4_^+^ and 7.5 mL for NO_3_^-^ analysis) was extracted after the sediment had settled (T = 0 minutes), and after both 3 (T = 3 minutes) and 10 minutes (T = 10 minutes). The initial 0–3 minutes flux represented a “physical” disturbance event, while the 3–10 minutes flux reflected a “biogeochemical” flux.

At each reach during four sampling events in spring 2016 (March-May), 20 random patches were selected and 10 mL of fine surficial sediment (top 2 cm of stream bottom) was collected with a stainless-steel scoop. Ambient water samples (grab samples taken from the downstream end of each reach) were also obtained at all sites, filtered (0.22 μm mixed cellulose ester membrane filters), and transported back to the laboratory and stored at -20°C. NH_4_^+^ and NO_3_^-^ analysis was conducted subsequently, using the method described below. Sediments collected from each random patch were transferred into 50 mL tubes and mixed with 35 mL stream water (Fig 2). For NH_4_^+^ analysis, 2.5 mL water was extracted (T = 0), and again after 3 (T = 3) and 10 minutes (T = 10). We equated the initial 0-3minute flux to physical disturbance events (e.g., sediments disturbed by a rising flood flows). The 3-10-minute flux was then equated to a biogeochemical flux which could mimic the movement of N between the water column and sediment layers due to biogeochemical processes. Based on a pilot study (http://dx.doi.org/10.17632/r2tt9gxkt2.1#file-56895ff4-2cd6-4326-9180-a749a9f98659), our 2 sampling periods reflected the time required for sediment particles to settle (T = 0-3min) and where water temperature would not be affected by air temperature (e.g., reflecting temperature effects on biogeochemical processes; T = 3–10 min). The 2.5 mL water samples for NH_4_^+^ analysis were added to 10 mL working reagent (containing 2 l borate buffer, 10 mL sodium sulphite, and 100 mL ortho-phthalaldehyde solution) in a separate vial and analysed using fluorometric methods [43]. An additional 7.5 mL water sample was filtered (0.22 μm mixed cellulose ester membrane filters), transported back to the laboratory and stored at -20°C. Subsequently, samples were thawed and NO_3_^-^ concentrations determined using ion chromatography. Due to the field-based nature of these assays, a few samples were not suitable for analysis, resulting in 12–20 replicates per reach with a final sample size of 158 successful assays completed.

### Sediment analysis

Sediment grain size analysis was carried out across all sites. Distributions were determined from 5 separate 10 g benthic sediment subsamples collected from both the restored and unrestored reaches of the study streams. Samples were dried (>24 hours at 60°C), weighed and sieved to separate coarse (>1 mm) and fine sediment (<1 mm). Sediment was dispersed into a Malvern Mastersizer 2000 granulometer and examined for average particle size. This procedure was repeated three times for each subsample. Samples were classified as either sand (0.063–2 mm), silt (0.004–0.063 mm), or clay (<0.004 mm).

After measuring N fluxes, sediment samples were mixed with 10 mL methanol for 1 minute and left in the dark for an hour to extract Chl-*a*. A 1.5 mL of the supernatant was transferred into an eppendorf tube and centrifuged for one minute at 3000 rpm. The absorbance of the sample was measured at 665 and 750 mm Abs to account for Chl-*a* extracted and background turbidity [44]. Chl-*a* concentrations were calculated and expressed as μg Chl-*a* g^-1^ dry weight using the following equation:

$$\frac{13.9\left[ABS_{665}-ABS_{750}\right]*vol\;extracted\;\left(mL\right)}{Sediment\;mass\;\left(g\right)}$$

For % total organic matter (TOM) sediment samples used for the Chl-*a* measurement were dried in an oven at 60°C for 24 hours. Samples were subsequently transferred into crucibles and weighed prior to and after ashing at 550°C for 6 hours. TOM was measured as a percentage of weight loss on ignition, and did not include the TOM associated with the extracted Chl-*a*.

### Data analysis

N fluxes were derived from the following equation:

$$\frac{[N_{2}]-\left[N_{1}\right]}{A*\left[t_{2}-t_{1}\right]}$$

where N_2_ and N _1_ refers to the NH_4_^+^ and NO_3_^-^ concentrations at t_2_ and t_1_, respectively; A is the surface area of the sediment surface (m^2^) and t_2_ –t_1_ = the time (sec) between the subsequent (t_2_) and previous (t_1_) water samples. NH_4_^+^ and NO_3_^-^ fluxes are expressed as μg N / (m^-2^ * sec). A positive flux indicates the movement of N from the sediment into overlying waters and a negative flux defines the movement of N from overlying waters into the sediment.

Average NH_4_^+^ and NO_3_^-^ concentrations and fluxes, Chl-*a* concentrations, and % TOM were compared between restored and unrestored sites on each river and between rivers using a 2-way ANOVA on ranks followed by a Tukey’s post-hoc test due to the lack of normality and non-equal variance in our datasets. Regression analyses were used to determine relationships between N water concentrations, Chl-*a*, % TOM and N fluxes. All statistics were performed using SigmaPlot 14.0.

## Results

### N water concentrations

NH_4_^+^ concentrations were highly variable across rivers (Table 2). Average concentrations at restored sites ranged from 36 μg L^-1^ to 731.7 μg L^-1^ and at unrestored sites from 28.0 μg L^-1^ to 290.5 μg L^-1^. However site-specific ranges were much greater, 8.3 μg L^-1^ to 1022 μg L^-1^ (Table 2). Concentrations were significantly different amongst rivers (F_4,171_ = 75.80; *p*<0.001), and significantly greater at restored reaches (F_4,171_ = 28.26; *p*<0.001). There was also a significant interaction between river and restoration (F_1,171_ = 18.65; *p*<0.001), although this was mainly due to the elevated concentrations at the Brent.

**Table 2:** A summary of ranges and averages (N = 20) of stream water NH_4_^+^ (μg L^-^) and NO_3_^-^ (mg L^-1^) concentrations in restored and unrestored reaches of London rivers during the spring months of 2016. Values in parenthesis are one standard error. Significant differences between restored and unrestored reaches are in bold; difference among rivers are indicated by letter groupings.

River	Restoration	NH_4_^+^ range	NH_4_^+^ average	NO_3_^-^ range	NO_3_^-^ average
Ravensbourne	Restored	37.3–438.8	146.3^a^ (33.7)	6.6–15.6	12.4^a^ (0.7)
	Unrestored	38.0–406.8	151.0^a^ (32.2)	8.8–17.0	12.5^a^ (0.7)
Pool	Restored	53.7–536.8	141.3^a,b^ (24.0)	7.7–16.3	13.0^b^ (0.5)
	Unrestored	53.5–266.9	115.0^a,b^ (11.8)	8.4–15.2	12.6^b^ (0.5)
Wandle	Restored	8.3–103.5	36.0^c^ (6.1)	16.5–27.7	**23.7**^**a,b**^ **(0.7)**
	Unrestored	11.3–103.2	28.0^c^ (4.8)	24.3–29.3	**26.4**^**a,b**^ **(0.3)**
Hogsmill	Restored	47.9–146.3	79.5^b,c^ (7.4)	14.3–28.2	22.7^a,b^ (0.6)
	Unrestored	31.1–106.2	56.5^b,c^ (5.4)	21.2–26.9	23.3^a,b^ (0.3)
Brent	Restored	241.8–1022	**731.7**^**d**^ **(84.3)**	7.3–15.3	9.6^a,b^ (0.6)
	Unrestored	202.0–471.0	**290.5**^**d**^ **(21.8)**	6.3–13.6	9.6^a,b^ (0.7)

Average NO_3_^-^ site concentrations at restored sites ranged from 9.6 mg L^-1^ to 23.7 mg L^-1^ whilst those at unrestored sites ranged from 9.6 mg L^-1^ to 26.4 mg L^-1^ (Table 2). NO_3_^-^ concentrations differed significantly between rivers (F_4.170_ = 282.94; *p*<0.001), but were not influenced by restoration (F_1.170_ = 2.71; *p* = 0.10). No significant interactions were found between rivers and restoration (F_1.170_ = 2.34; *p* = 0.06).

### N flux across the sediment-water interface

**Fig 3:**
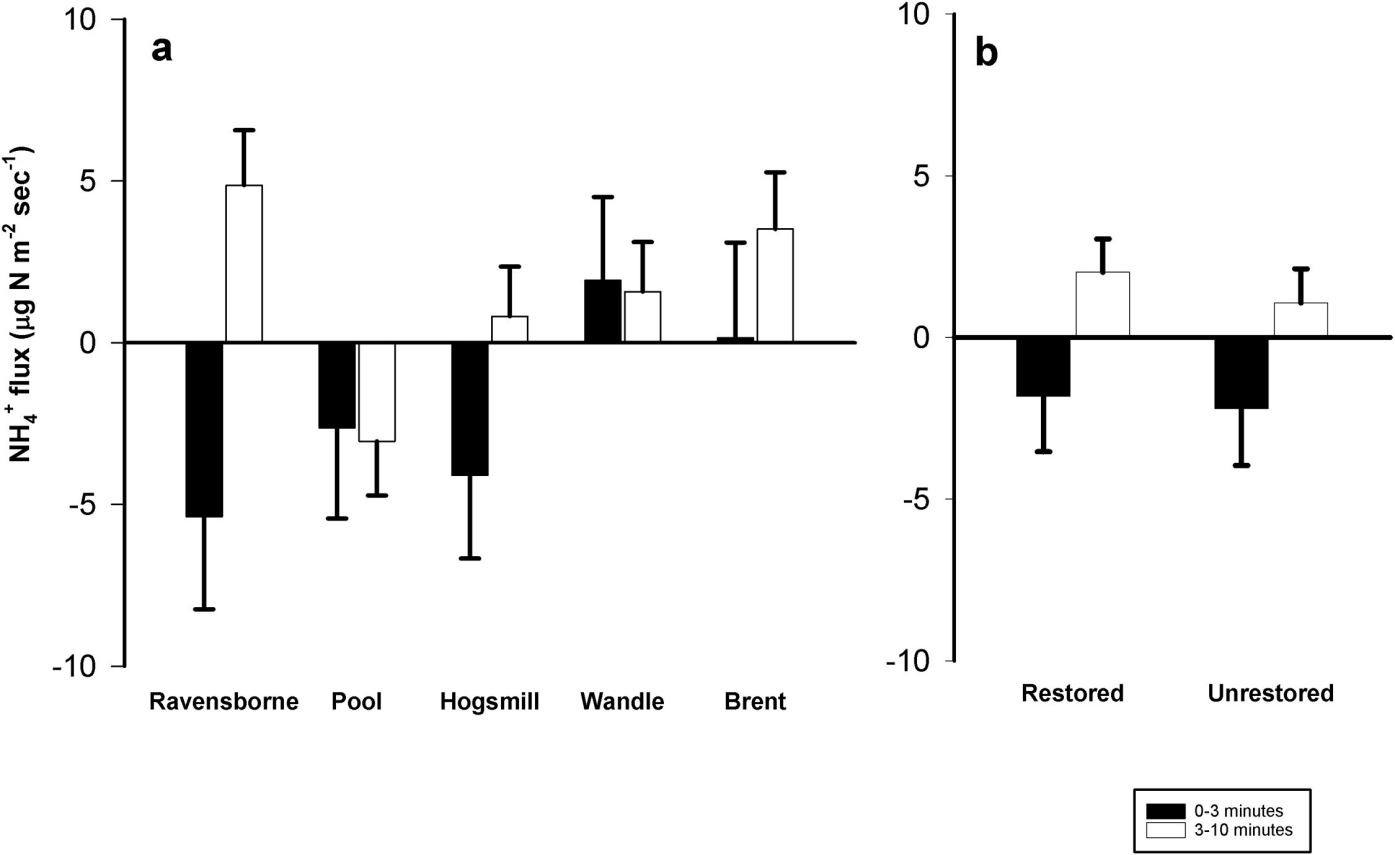
**Average NH_4_^+^ fluxes (μg N m^-2^ sec^-1^) among (a) the study rivers (restored and unrestored combined) and between (b) the combined restored and unrestored reaches from all London rivers.** Columns represent average values (N = 12–20) + one standard error. Both physical disturbance (T = 0–3 minutes) and biogeochemical activity (T = 3–10 minutes) are presented in each panel. There was no significance different between river NH_4_^+^ fluxes over the 0–3 minutes period, nor between restored or unrestored reaches at both 0–3 and 3–10 minutes. Rivers with different letters show significant differences in fluxes over the 3–10 minutes. Positive flux values represent uptake/removal of nutrients from the water column and negative flux values represent release of nutrients from the sediment (regeneration).

**Fig 4:**
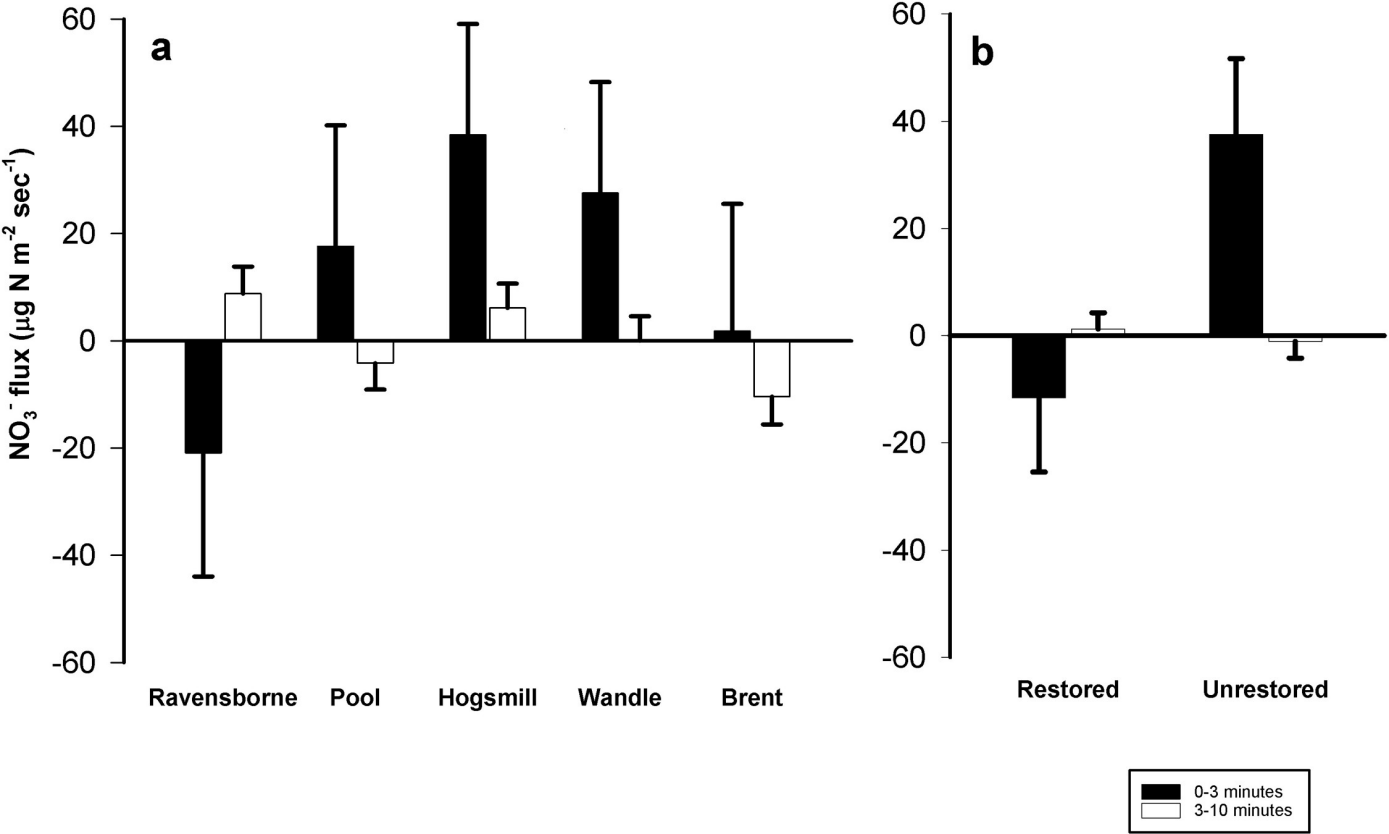
**Average NO_3_^-^ fluxes (μg N m^-2^ sec^-1^) among a) the study rivers (restored and unrestored combined) and between (b) the combined restored and unrestored reaches from all London rivers.** Columns represent average values (N = 12–20) + one standard error. Both physical disturbance (T = 0–3 minutes) and biogeochemical activity (T = 3–10 minutes) are presented in each panel. There was no significant difference in NO3^-^ fluxes between rivers. However, there was a significant regeneration of NO_3_^-^ from sediment in unrestored sites over the 0–3 minutes period, but not difference between fluxes at 3–10 minutes. Positive flux values represent uptake/removal of nutrients from the water column and negative flux values represent release of nutrients from the sediment (regeneration).

Across the entire experiment both NH_4_^+^ and NO_3_^-^ fluxes showed uptake and regeneration, and we found no constant patterns in magnitude or direction amongst these measurements (Table 3 and Figs 3 and 4). Average NH_4_^+^ fluxes for 0–3 minutes across all rivers ranged from -8.9 to 3.4 μg N m^-2^ sec^-1^, and did not differ significantly (F_4,158_ = 1.25; *p* = 0.29) (Fig 3A and Table 3). There were no significant differences in 0–3 minutes NH_4_^+^ fluxes between restored and unrestored sites (F_1,158_ = 0.02; *p* = 0.88) (Fig 3B). NH_4_^+^ fluxes for 3–10 minutes showed both uptake and regeneration (-7.1 to 7.5 μg N m^-2^ sec^-1^) and were significantly different (F_4,158_ = 3.20; *p* = 0.015) (Fig 3A and Table 3). However, restoration had no influence on 3–10 minutes fluxes (F_1,158_ = 0.42; *p* = 0.52; Fig 3B).

**Table 3:** A summary of N flux averages (μg N m^-2^ sec-^1^) of site-specific measurement (N = 12–20). Values in parenthesis are one standard error. Significant differences between restored and unrestored reaches are in bold. Positive flux values represent uptake/removal of nutrients from the water column and negative flux values represent release of nutrients from the sediment (regeneration). Uptake is shaded brown and regeneration is shade blue. Overall, there were no constant patterns in the magnitude or direction of flux among all measurements.

River	Restoration	0–3 min NH_4_^+^ flux	3–10 min NH_4_^+^ flux	0–3 min NO_3_^-^ flux	3–10 min NO_3_^-^ flux
Ravensbourne	Restored	-8.9 (5.9)	2.2 (1.4)	-32.6 (24.1)	16.0 (5.6)
	Unrestored	-1.9 (4.7)	7.5 (5.8)	-9.3 (21.7)	1.6 (5.6)
Pool	Restored	1.0 (2.1)	**1.0 (0.6)**	28.4 (28.1)	-13.5 (6.8)
	Unrestored	-6.3 (5.4)	**-7.1 (5.0)**	6.7 (28.8)	5.2 (4.9)
Wandle	Restored	3.3 (1.3)	1.6 (0.8)	0.3 (34.8)	-4.8 (7.1)
	Unrestored	0.5 (1.6)	1.5 (0.3)	54.6 (40.8)	4.9 (9.1)
Hogsmill	Restored	-2.9 (2.8)	0.2 (0.9)	**-20.9 (19.2)**	14.8 (8.0)
	Unrestored	-5.4 (3.2)	1.5 (0.9)	**97.7 (44.8)**	-2.6 (5.1)
Brent	Restored	-1.7 (6.7)	5.0 (2.3)	**-33.6 (19.0)**	-6.4 (3.2)
	Unrestored	2.0 (4.0)	2.0 (1.2)	**37.0 (22.4)**	-14.4 (7.2)

NO_3_^-^ fluxes for 0–3 minutes across all sites ranged from -33.6 to 97.8 μg N m^-2^ sec^-1^ (Table 3). (There were significant differences between restored and unrestored sites, with uptake in the restored sites and regeneration in the unrestored sites (F_1,158_ = 6.14; *p* = 0.014; Fig 4B). However, there were no differences among rivers (F_4,158_ = 1.1; *p* = 0.36; Fig 4A and Table 3). Average NO_3_^-^ fluxes for 3–10 minutes across all sites ranged from -14.4 to 16.0 μg N m^-2^ sec^-1^ (Table 3), with no significant differences found between restored and unrestored reaches (F_1,158_ = 0.28; *p* = 0.60; Fig 4B and Table 3) or amongst study rivers (F_4,158_ = 2.38; *p* = 0.05; Fig 4A and Table 3).

### Relationship between sediment grain size, Chl-*a*, % TOM and flux

Sediment grain size amongst all sampling locations varied little and was predominantly sand (Table 4). Average Chl-*a* concentrations at restored sites ranged from 0.3 to 1.9 μg g^-1^, whilst those at unrestored sites ranged from 0.4 to 0.7 μg g^-1^ (Table 4). There were significant differences for Chl-*a* amongst rivers (F_4,148_ = 2.95; *p* = 0.02), with the Wandle differing from the Hogsmill and Pool and there was also a significant difference between the restored and unrestored reaches at the Wandle (*p* = 0.003) (Table 4). However, restoration did not have an overall effect on Chl-*a* concentrations between restored and unrestored reaches (F_1,148_ = 2.52; *p* = 0.12). Average % TOM ranged from 18.54 to 30.83% across restored and unrestored reaches (Table 4), but did not differ significantly amongst rivers (F_4,158_ = 2.22; *p* = 0.070). However, % TOM was also significantly higher at the restored compared to unrestored site on the Wandle, and at the unrestored site compared to the restored site on the Pool, but not between the two reaches from the other rivers (Table 4, F_4,158_ = 0.80; *p* = 0.37). Across our regression analyses, there were no significant relationships found between N water concentrations, % TOM and Chl-*a* to either NH_4_^+^ and NO_3_^-^ fluxes associated with disturbance or biogeochemical activity (i.e., R^2^<0.03; *p*>0.05).

**Table 4:** A summary of the average (N = 12–20) sediment grain size, Chl-*a*, and percentage total organic matter. Values in parenthesis are one standard error. Significant differences between restored and unrestored reaches are in bold; difference among rivers are indicated by letter groupings.

River	Reach	Sediment grain size (% sand)	Chl-*a* (μg g^1^)	Total organic matter (%)
Ravensbourne	Restored	93 (0.4)	0.6^a,b^ (0.1)	20.6 (1.7)
	Unrestored	94 (0.6)	0.5^a,b^ (0.1)	19.4 (2.2)
Pool	Restored	96 (0.2)	0.3^a^ (0.1)	**18.5 (2.2)**
	Unrestored	96 (0.6)	0.6^a^ (0.1)	**26.6 (2.8)**
Wandle	Restored	91 (0.6)	**1.9**^**b**^ **(0.9)**	**30.8 (4.5)**
	Unrestored	97 (0.1)	**0.7**^**b**^ **(0.2)**	**21.1 (2.4)**
Hogsmill	Restored	92 (1.0)	0.6^a^ (0.1)	27.1 (2.6)
	Unrestored	93 (0.1)	0.4^a^ (0.1)	27.0 (1.5)
Brent	Restored	96 (0.3)	0.8^a,b^ (0.2)	28.4 (3.8)
	Unrestored	98 (0.2)	0.4^a,b^ (0.1)	23.5 (2.7)

## Discussion

Results from this study indicate that restoration in these streams had no consistent overall effect on NH_4_^+^ and NO_3_^-^ uptake or regeneration rates from sediments in our experimental setups (Fig 3 and 4 and Table 3). This may not be surprising given the highly urban nature of London rivers, in which nutrient loading and sediment N saturation are likely to be offsetting any N removal associated with restoration [3,18], and also due to the varied nature of restoration actions take in each river (Table 1). However, our uptake values are in line with those reported across a range of stream types for NH_4_^+^ flux [14,45] and NO_3_^-^ flux [14,16,46]. Furthermore, our values are similar to those seen in urban systems [11,14,14,47] and restored sites [48,49]. Much like the “field of dreams” hypothesis [40], (i.e. the assertion that habitat enhancement will improve biotic integrity [32,50] in-channel restoration measures focused on improving habitat and flow might be expected to accrue additional benefits associated with overall N dynamics (e.g., metabolism, assimilation and transport). This could be the case at restored reaches of the Ravensbourne, Pool, Wandle and Hogsmill where in-stream berms and cobbles have been deployed to re-naturalize flows (Table 1), which may simultaneously stimulate sediment deposition and facilitate N assimilation. Given the extent of N loading among the study rivers, coupled with the varying timescales over which ecological and chemical indices respond to restoration, it is not surprising that we had equivocal results. This is further supported by previous studies, which have found variable responses of restoration on N dynamics [39,51,52]. In our study there was insufficient evidence to suggest that restoration is leading to improvements in either water quality (Table 2) or N flux (Figs 3 and 4 and Table 3). Even for projects where ecological characteristics may positively respond to reach-scale restoration, it is likely that poor water quality throughout catchments may impinge upon any significant improvements; conditions which we feel account for the results in this study. However, whilst our observation of a lack of a “restoration effect” was consistent, a caveat is that the results come from 5 unique streams in urban London, with data collected at the patch scale.

Across all the study sites, restoration practices did not lead to significant reductions in NH_4_^+^ or NO_3_^-^ concentrations (Table 3). NH_4_^+^ concentrations varied widely across sites, aligning with previously reported values observed in London tributaries [23,24]. This highlights the heavily impacted nature of London rivers upon which multiple stressors are acting. In contrast, NO_3_^-^ concentrations differed significantly from previous studies, highlighting a ~50% rise in concentrations >20 mg L^-1^ at the Wandle and Hogsmill, and a concentration decrease of a similar magnitude at the Brent (Fig 3). These concentrations are comparable to previously reported values along the Thames catchment [21,53]; these are often lower than other urban rivers of Europe which can exceed 100 mg L^-1^ [54,55]. Higher concentrations of NO_3_^-^ versus NH_4_^+^ were observed across all sites, which may be attributed to nitrification processes occurring in-stream and uptake distances that are shorter for NH_4_^+^ than NO_3_^-^ [16,30,56]. Previous links have been made between inorganic N inputs in headwater streams and rapid N removal which highlights the potential for removal or transformation across small temporal and spatial scales [57]. However, this is not the case in London streams, and is likely to be due to N sediment saturation and continuous pollution loading [1].

Initially, we were surprised that overlying NH_4_ and NO_3_^-^ concentrations did not correspond with uptake or regeneration fluxes. Several studies have reported positive relationships between N concentrations and uptake in urban streams resulting from restoration activities [15,16,33,37,46]. However, this differs from other studies which highlight the role of biogeochemical transformations in triggering NO_3_^-^ reduction to NH_4_^+^ and N_2_ in anaerobic sediments [29,58]. The highly urban nature of our study streams, combined with potential N removal and transformations (ammonification, nitrification and denitrification) across the sediment-water interface, may explain these differences. This is supported by previous studies which have identified that urban cover >20% can hinder stream responses to restoration [3,39]. Percent urban cover at sites used for this study far exceed these values, ranging from 47–69% (Table 1). Increases in N concentration can further reduce the capacity of streams to retain and transform N inputs, leading to a reduction in biotic uptake and denitrification [18,56]. This supports a lack of relationship observed between Chl-*a* and N flux, which differs from other studies linking Chl-*a* to N concentrations, % TOM and suspended sediments [21]. Significant NO_3_^-^uptake rates were recorded at the Ravensbourne, Hogsmill and Brent following physical disturbances (e.g., 0–3 minutes treatment). This may be attributed to NO_3_^-^ uptake and assimilation following disturbances [25,37,59,60]. However, no significant relationship was observed for the biogeochemical flux, thus it is difficult to determine any restoration success related to N dynamics. Biogeochemical processing of flux between N dynamic and ambient water warrants further research, specifically looking at nutrient uptake limitations and the relationship between N supply and biological demand [61].

Our approach using N flux assay in small chambers focuses on processes which occur at the sediment-water interface. This approach may provide an appropriate scale for evaluating a wide range of restoration practices which occur in urban rivers because of its patch-scale focus. It is important to acknowledge that there are limitations to this approach, as it is difficult to extrapolate to reach-scale N flux, which are more commonly reported in the literature [11]. This method is easier and affordable to implement compared to catchment- and reach-scale methods, which require long-term synoptic monitoring or tracer techniques [18,45,56,62]. However, comparison with other research projects reporting spiralling is not straightforward. Therefore, methods for adapting our approach to allow for upscaling to evaluate impacts to downstream systems needs further development. In addition, experiments to evaluate temporal changes between physical and biological processes, especially related to potential temperature-mediated effects, are required. Despite these issues, our results do provide evidence to show that river restoration in highly urban streams is unlikely to support predictable changes in N dynamics without greater understanding of site-specific factors which affect disturbance and biogeochemical-associated flux [48,62].

### Future management approaches

Reach-scale restoration did not influence N flux across the sediment-water interface at our study sites. This should not necessarily be perceived as a restoration failure, but an opportunity to examine restoration responses across different spatial and temporal scales. Given the small size of restored reaches within this study and urban catchments which experience a myriad of multiple stressors [2,3,5,8], it is perhaps not surprising that no significant N-specific benefits were accrued. In combination with the delayed response of pollutants to restoration, these highlight the need for larger scale restoration studies to be undertaken over prolonged timescales. Whilst many projects examine the fate of accumulated N in middle and downstream reaches [14,18,63], few focus on targeting N inputs in headwater streams [56,59]. Headwater reaches are highly susceptible to nutrient loading from urban land, therefore restoration could provide widespread potential to mitigate against eutrophication associated with N loading [27,29]. Selecting restoration sites in headwaters based on optimal dimensions between area, size, discharge and velocity can positively influence uptake N metrics [64]. This will help to create a buffer for downstream environments where an increasing urban gradient is likely to reduce N removal capacity.

The range of restoration practices applied to our study sites did not produce consistent results, therefore additional restoration practices could potentially improve the condition of these urban rivers. For example, Sustainable Urban Drainage Systems [65] have the potential to remove N, through the use of wetlands, swales, and attenuation ponds across sensitive catchment areas. Stream daylighting is also increasingly being adopted as a restoration strategy to increase hyporheic exchange and eliminate excess N in the presence of bioavailable carbon [66]. Integrating vegetative structures can help to restore natural flow regime resulting from channelization, whilst combatting problems associated with thermal stress [1,67]. Future restoration projects should seek to determine how habitat alterations and hydrological regime can stimulate N uptake whilst building resilience to disturbance events [34]. Irrespective of these management options, rivers in London and other similar cities still have a legacy of widespread misconnections which are contributing to significant amounts of effluent entering into these urban rivers.

## Conclusions

This study sought to determine whether river restoration activities could influence N dynamics of degraded rivers in London. This small-scale approach highlighted the dynamic nature of N processing occurring within urban river reaches. Results highlighted that NH_4_^+^ concentrations were significantly higher at restored sites than unrestored sites, whilst NO_3_^-^ concentrations did not differ between reaches. Overall, restoration did not significantly alter NH_4_^+^ or NO_3_^-^ fluxes. This suggests that a synergy of geomorphic and biogeochemical processes, including natural and artificial stream morphology, stream bed characteristics, availability of nutrients, and temperature are also likely to be influencing N processing, which need further investigation.

There is a critical need to better understand the mechanisms controlling the inputs, processing and transformations of NH_4_^+^ and NO_3_^-^ into urban river systems. This is particularly true for the highly urbanised system found in megacities like London, which far exceed impervious cover value observed in other cities. Future research should focus on incorporating combined on-site outfall identification work and tracer studies to determine the source, saturation concentrations and fate of N. Supporting studies should examine other environmental variables which may be influencing flux dynamics. Sediment-water nutrient interactions have historically been overlooked in restoration studies in favour of aesthetic, hydrological and biological improvements. If the overall aim of river restoration is to improve ecosystem function, these factors should be considered as interacting components to maximise the chance of ecosystem recovery and build resilience to future perturbations.

## References

[1] Paul MJ, Meyer JL. Streams in the Urban Landscape. Annu Rev Ecol Syst. 2001;32(1):333–65.

[2] Meyer JL, Paul MJ, Taulbee WK. Stream ecosystem function in urbanizing landscapes. J North Am Benthol Soc. 2005;24(3):602–12.

[3] Walsh CJ, Roy AH, Feminella JW, Cottingham PD, Groffman PM, Morgan RP. The urban stream syndrome: current knowledge and the search for a cure. J North Am Benthol Soc. 2005;24(3):706–23.

[4] Booth DB, Roy AH, Smith B, Capps KA. Global perspectives on the urban stream syndrome. Freshw Sci. 2016;35(1):412–20.

[5] Vietz GJ, Walsh CJ, Fletcher TD. Urban hydrogeomorphology and the urban stream syndrome. Prog Phys Geogr. 2016;40(3):480–92.

[6] Vörösmarty CJ, McIntyre PB, Gessner MO, Dudgeon D, Prusevich A, Green P, et al Global threats to human water security and river biodiversity. Nature. 2010;467(7315):555–61. 10.1038/nature09440 20882010

[7] Vilmin L, Mogollón JM, Beusen AHW, Bouwman AF. Forms and subannual variability of nitrogen and phosphorus loading to global river networks over the 20th century. Glob Planet Change. 2018;163:67–85.

[8] Chadwick MA, Dobberfuhl DR, Benke AC, Huryn AD, Suberkropp K, Thiele JE. Urbanization affects stream ecosystem function by altering hydrology, chemistry and biotic richness. Ecol Appl. 2006;16(5):1796–807. 17069372 10.1890/1051-0761(2006)016[1796:uasefb]2.0.co;2

[9] Harrison MD, Groffman PM, Mayer PM, Kaushal SS, Newcomer TA. Denitrification in Alluvial Wetlands in an Urban Landscape. J Environ Qual. 2011;40(2):634 21520770 10.2134/jeq2010.0335

[10] Kaye JP, Groffman PM, Grimm NB, Baker LA, Pouyat R V. A distinct urban biogeochemistry? Trends Ecol Evol. 2006;21(4):192–9. 10.1016/j.tree.2005.12.006 16701085

[11] Reisinger AJ, Groffman PM, Rosi-Marshall EJ. Nitrogen-cycling process rates across urban ecosystems. FEMS Microbiol Ecol. 2016;92(12):fiw198. 10.1093/femsec/fiw198 27660607

[12] Kaushal SS, Belt KT. The urban watershed continuum: evolving spatial and temporal dimensions. Urban Ecosyst. 2012;15(2):409–35.

[13] Rueda J, Camacho A, Mezquita F, Hernández R, Roca JR. Effect of Episodic and Regular Sewage Discharges on the Water Chemistry and Macroinvertebrate Fauna of a Mediterranean Stream. Water Air Soil Pollut. 2002;140(1):425–44.

[14] Bernot MJ, Dodds WK. Nitrogen Retention, Removal, and Saturation in Lotic Ecosystems. Ecosystems. 2005;8(4):442–53.

[15] Pennino MJ, Kaushal SS, Beaulieu JJ, Mayer PM, Arango CP. Effects of urban stream burial on nitrogen uptake and ecosystem metabolism: implications for watershed nitrogen and carbon fluxes. Biogeochemistry. 2014;121(1):247–69.

[16] Grimm NB, Sheibley RW, Crenshaw CL, Dahm CN, Roach WJ, Zeglin LH. N retention and transformation in urban streams. J North Am Benthol Soc. 2005;24(3):626–42.

[17] Mekonnen MM, Hoekstra AY. Global Gray Water Footprint and Water Pollution Levels Related to Anthropogenic Nitrogen Loads to Fresh Water. Environ Sci Technol. 2015;49(21):12860–8. 10.1021/acs.est.5b03191 26440220

[18] Mulholland PJ, Helton AM, Poole GC, Hall RO, Hamilton SK, Peterson BJ, et al Stream denitrification across biomes and its response to anthropogenic nitrate loading. Nature. 2008;452(7184):202–5. 10.1038/nature06686 18337819

[19] Craig LS, Palmer MA, Richardson DC, Filoso S, Bernhardt ES, Bledsoe BP, et al Stream restoration strategies for reducing river nitrogen loads. Front Ecol Environ. 2008;6(10):529–38.

[20] Kaushal SS, Groffman PM, Band LE, Elliott EM, Shields CA, Kendall C. Tracking Nonpoint Source Nitrogen Pollution in Human-Impacted Watersheds. Environ Sci Technol. 2011;45(19):8225–32. 10.1021/es200779e 21830824

[21] Neal C, Hilton J, Wade AJ, Neal M, Wickham H. Chlorophyll-a in the rivers of eastern England. Sci Total Environ. 2006;365(1–3):84–104. 10.1016/j.scitotenv.2006.02.039 16626783

[22] Davies G. A water quality analysis of the River Lee and major tributaries within the perimeter of the M25, from Waltham Abbey to Bow Locks [Internet]. 2011 [cited 2018 Jun 18]. Available from: http://www.thames21.org.uk/Downloads/A water quality analysis of the River Lea and major tributaries within the perimeter of the M25, from Waltham Abbey to Bow Locks -Thames21.pdf

[23] Ammonium in rivers—European Environment Agency [Internet]. 2017 [cited 2018 Jun 18]. Available from: http://www.eea.europa.eu/data-and-maps/explore-interactive-maps/wise-soe-ammonium-in-rivers

[24] Pecorelli: J. An audit of the surface water outfalls in parts of the River Brent Catchment -’Outfall Safari’ [Internet]. London, UK; 2017 [cited 2018 Jun 18]. Available from: www.zsl.org/conservation/regions/uk-europe/london’s-rivers

[25] Valett HM, Morrice JA, Dahm CN, Campana ME. Parent lithology, surface-groundwater exchange, and nitrate retention in headwater streams. Limnol Oceanogr. 1996;41(2):333–45.

[26] Maksymowska-Brossard D. P-JH. Seasonal variability of benthic NH4+ release in the surface sediments of the Gulf Gdańsk (Southern Baltic Bay). Oceanologia. 2001;43:113–36.

[27] Wu Y, Li T, Yang L. Mechanisms of removing pollutants from aqueous solutions by microorganisms and their aggregates: A review. Vol. 107, Bioresource Technology. Elsevier; 2012 p. 10–8. 10.1016/j.biortech.2011.12.088 22257855

[28] Lijklema L, Koelmans AA, Portielje R. Water Quality Impacts of Sediment Pollution and the Role of Early Diagenesis. Water Sci Technol. 1993;28(8–9).

[29] Clavero V, Izquierdo J, Fernández J, Niell F. Seasonal fluxes of phosphate and ammonium across the sediment-water interface in a shallow small estuary (Palmones River, southern Spain). Mar Ecol Prog Ser. 2000;198:51–60.

[30] Kaushal SS, Groffman PM, Mayer PM, Striz E, Gold AJ. Effects of stream restoration on denitrofication in an urbanizing watershed. Ecol Appl. 2008;18(3):789–804. 18488635 10.1890/07-1159.1

[31] Bernhardt ES, Palmer MA, Allan JD, Alexander G, Barnas K, Brooks S, et al Synthesizing U.S. River Restoration Efforts. Science (80-). 2005;308(5722):636–7. 10.1126/science.1109769 15860611

[32] Bernhardt ES, Palmer MA. Restoring streams in an urbanizing world. Freshw Biol. 2007;52(4):738–51.

[33] Roberts BJ, Mulholland PJ, Houser JN. Effects of upland disturbance and instream restoration on hydrodynamics and ammonium uptake in headwater streams. J North Am Benthol Soc. 2007;26:38–53.

[34] Wohl E, Lane SN, Wilcox AC. The science and practice of river restoration. Water Resour Res. 2015;51(8):5974–97.

[35] Kasahara T, Hill AR. Hyporheic exchange flows induced by constructed riffles and steps in lowland streams in southern Ontario, Canada. Hydrol Process. 2006;20(20):4287–305.

[36] Bukaveckas PA. Effects of channel restoration on water velocity, transient storage, and nutrient uptake in a channelized stream. Environ Sci Technol. 2007;41(5):1570–6. 17396643 10.1021/es061618x

[37] Hines SL, Hershey AE. Do channel restoration structures promote ammonium uptake and improve macroinvertebrate-based water quality classification in urban streams? Inl Waters. 2011;1(2):133–45.

[38] Webster JR, Mulholland PJ, Tank JL, Valett HM, Dodds WK, Peterson BJ, et al Factors affecting ammonium uptake in streams—an inter-biome perspective. Freshw Biol. 2003;48(8):1329–52.

[39] Smith B, Chadwick MA. Litter decomposition in highly urbanized rivers: influence of restoration on ecosystem function. Fundam Appl Limnol / Arch für Hydrobiol. 2014;185(1):7–18.

[40] Cook H. ‘An Unimportant River in the Neighbourhood of London’: The Use and Abuse of the River Wandle. Lond J. 2015;40(3):225–43.

[41] Pike T, Bedford C, Davies B, Brown D. A Catchment Plan for the River Wandle Prepared by the Wandle Trust on behalf of the communities and stakeholders of the Wandle Catchment [Internet]. London, UK; 2014 [cited 2018 Jun 18]. Available from: https://www.wandletrust.org/wp-content/uploads/2014/12/Wandle_Catchment_Plan_-_Sept_2014_-_full_document.pdf

[42] River Restoration Centre. River Brent at Tokyngton Park: Techniques: Re-meandering, backwater creation, de-culverting [Internet]. 2008 [cited 2017 Nov 26]. Available from: https://www.therrc.co.uk/case_studies/tokyngton park.pdf

[43] Holmes RM, Aminot A, Kerouel R, Hooker BA, Peterson BJ. A simple and precise method for measuring ammonium in marine and freshwater ecosystems. Can J Fish Aquat Sci. 1999;56:1801–8.

[44] Marker AFH. Chlorophyll a SCA method revision [Internet]. Cambridgeshire PE17 2LS; 1994 [cited 2016 May 26]. Available from: http://nora.nerc.ac.uk/id/eprint/509591/1/N509591CR.pdf

[45] Tank JL, Martí E, Riis T, von Schiller D, Reisinger AJ, Dodds WK, et al Partitioning assimilatory nitrogen uptake in streams: an analysis of stable isotope tracer additions across continents. Ecol Monogr. 2018;88(1):120–38.

[46] Hall RO, Tank JL, Sobota DJ, Mulholland PJ, O’Brien JM, Dodds WK, et al Nitrate removal in stream ecosystems measured by 15N addition experiments: Total uptake. Limnol Oceanogr. 2009;54(3):653–65.

[47] O’Brien JM, Dodds WK, Wilson KC, Murdock JN, Eichmiller J. The saturation of N cycling in Central Plains streams:15N experiments across a broad gradient of nitrate concentrations. Biogeochemistry. 2007;84(1):31–49.

[48] Klocker CA, Kaushal SS, Groffman PM, Mayer PM, Morgan RP. Nitrogen uptake and denitrification in restored and unrestored streams in urban Maryland, USA. Aquat Sci. 2009;71(4):411–24.

[49] Sudduth EB, Hassett BA, Cada P, Bernhardt ES. Testing the Field of Dreams Hypothesis: functional responses to urbanization and restoration in stream ecosystems. Ecol Appl. 2011;21(6):1972–88. 21939038 10.1890/10-0653.1

[50] Palmer MA, Ambrose RF, Poff NL. Ecological Theory and Community Restoration Ecology. Restor Ecol. 1997;5(4):291–300.

[51] Roni P, Hanson K, Beechie T. Global Review of the Physical and Biological Effectiveness of Stream Habitat Rehabilitation Techniques. North Am J Fish Manag. 2008;28(3):856–90.

[52] Filoso S, Palmer MA. Assessing stream restoration effectiveness at reducing nitrogen export to downstream waters. Ecol Appl. 2011;21:1989–2006. 21939039 10.1890/10-0854.1

[53] Halliday SJ, Skeffington RA, Wade AJ, Bowes MJ, Gozzard E, Newman JR, et al High-frequency water quality monitoring in an urban catchment: hydrochemical dynamics, primary production and implications for the Water Framework Directive. Hydrol Process. 2015;29(15):3388–407.

[54] Floury M, Usseglio-Polatera P, Ferreol M, Delattre C, Souchon Y. Global climate change in large European rivers: long-term effects on macroinvertebrate communities and potential local confounding factors. Glob Chang Biol. 2013;19(4):1085–99. 10.1111/gcb.12124 23504886

[55] Minaudo C, Meybeck M, Moatar F, Gassama N, Curie F. Eutrophication mitigation in rivers: 30 years of trends in spatial and seasonal patterns of biogeochemistry of the Loire River (1980–2012). Biogeosciences. 2015;12(8):2549–63.

[56] Peterson BJ, Wollheim WM, Mulholland PJ, Webster JR, Meyer JL, Tank JL, et al Control of nitrogen export from watersheds by headwater streams. Science. 2001;292(5514):86–90. 10.1126/science.1056874 11292868

[57] Alexander RB, Smith RA, Schwarz GE. Effect of stream channel size on the delivery of nitrogen to the Gulf of Mexico. Nature. 2000;403(6771):758–61. 10.1038/35001562 10693802

[58] Martens CS, Val Klump J. Biogeochemical cycling in an organic-rich coastal marine basin 4. An organic carbon budget for sediments dominated by sulfate reduction and methanogenesis. Geochim Cosmochim Acta. 1984;48(10):1987–2004.

[59] Simon KS, Chadwick MA, Huryn AD, Valett HM. Stream ecosystem response to chronic deposition of N and acid at the Bear Brook Watershed, Maine. Environ Monit Assess. 2010;171(1–4):83–92. 10.1007/s10661-010-1532-2 20535547

[60] Roley SS, Tank JL, Williams MA. Hydrologic connectivity increases denitrification in the hyporheic zone and restored floodplains of an agricultural stream. J Geophys Res. 2012 9;117:G00N04.

[61] Covino TP, Bernhardt ES, Heffernan JB. Measuring and interpreting relationships between nutrient supply, demand, and limitation. Freshw Sci. 2018;37(3):448–55.

[62] Sivirichi GM, Kaushal SS, Mayer PM, Welty C, Belt KT, Newcomer TA, et al Longitudinal variability in streamwater chemistry and carbon and nitrogen fluxes in restored and degraded urban stream networks. J Environ Monit. 2011;13(2):288–303. 10.1039/c0em00055h 21116542

[63] Alexander RB, Böhlke JK, Boyer EW, David MB, Harvey JW, Mulholland PJ, et al Dynamic modeling of nitrogen losses in river networks unravels the coupled effects of hydrological and biogeochemical processes. Biogeochemistry. 2009;93(1–2):91–116.

[64] Johnson ZC, Warwick JJ, Schumer R. Nitrogen retention in the main channel and two transient storage zones during nutrient addition experiments. Limnol Oceanogr. 2015;60(1):57–77.

[65] Zhou Q. A review of sustainable urban drainage systems considering the climate change and urbanization impacts. Water. 2014 4 22;6(4):976–92.

[66] Neale MW, Moffett ER. Re-engineering buried urban streams: Daylighting results in rapid changes in stream invertebrate communities. Ecol Eng. 2016;87:175–84.

[67] Kim YH, Ryoo SB, Baik JJ, Park IS, Koo HJ, Nam JC. Does the restoration of an inner-city stream in Seoul affect local thermal environment?. Theoretical and applied climatology. 2008 5 1;92(3–4):239–48.

